# Enhancement of Cisplatin Sensitivity in Human Cervical Cancer: Epigallocatechin-3-Gallate

**DOI:** 10.3389/fnut.2014.00028

**Published:** 2015-01-26

**Authors:** Ulkan Kilic, Kazim Sahin, Mehmet Tuzcu, Nazli Basak, Cemal Orhan, Birsen Elibol-Can, Ertugrul Kilic, Fikrettin Sahin, Omer Kucuk

**Affiliations:** ^1^Department of Medical Biology and Regenerative and Restorative Medicine Research Center (REMER), Faculty of Medicine, Istanbul Medipol University, Istanbul, Turkey; ^2^Department of Animal Nutrition, Faculty of Veterinary Medicine, Firat University, Elazıg, Turkey; ^3^Department of Biology, Faculty of Science, Firat University, Elazıg, Turkey; ^4^Department of Genetics and Bioengineering, Faculty of Engineering and Architecture, Yeditepe University, Istanbul, Turkey; ^5^Department of Medical Biology, Faculty of Medicine, Bezmialem Vakif University, Istanbul, Turkey; ^6^Department of Physiology, Faculty of Medicine, Istanbul Medipol University, Istanbul, Turkey; ^7^Winship Cancer Institute, Emory University, Atlanta, GA, USA

**Keywords:** cisplatin, epigallocatechin gallate, HeLa cells, human cervical cancer, sensitization

## Abstract

Cisplatin is one of the effective chemotherapeutics in the treatment of several types of cancers. However, in addition to the efforts against to its toxicity, the amelioration of cisplatin sensitivity is an important point in treatment of cervical cancer. To do so, additional substances such as epigallocatechin gallate (EGCG), a polyphenol in green tea, have been used in combination with chemotherapeutics. We aimed to investigate the possible molecular pathways to potentiate cervical cancer cell (HeLa) growth inhibition by combination therapy of cisplatin and EGCG. HeLa cells were treated with EGCG (25 μM), cisplatin (250 nM), and their combination for 24 h. Cell viability was determined by MTS Assay. We analyzed the expressions of NF-κB p65, COX-2, Nrf2, HO-1, p-mTOR, p-p70S6K1, p-4E-BP1, and p-Akt by Western blot analysis. Herein, we have demonstrated that EGCG works synergistic with cisplatin in inhibiting growth of cervical cancer cells. EGCG improved efficacy of cisplatin treatment in HeLa cells by regulating NFκB p65, COX-2, p-Akt, and p-mTOR pathways, whereas it increased the expression levels of Nrf2/HO-1 in combined therapy. Our observations revealed that EGCG increases the sensitization of cisplatin to cervical cancer cells by inhibiting cell survival and inducing apoptosis.

## Introduction

Chemotherapeutic agents, anti-neoplastic cytotoxic drugs, can greatly improve an individual’s chances of survival by impairing mitosis of fast-dividing cancer cells. Cisplatin (cis-diamminedichloroplatinum II, CDDP), an inorganic platinum compound, is one of the effective chemotherapeutic agents in the treatment of several types of cancer, including cervical carcinomas (1). As other anti-cancer drugs, the mechanism of cisplatin action occurs by induction of cell death through DNA damage. However, it has adverse side-effects such as nephrotoxicity, gastrointestinal toxicity, neurotoxicity, bone marrow toxicity, and ototoxicity (2). The common side-effects of cisplatin usage are generally induced by oxidative stress due to strong electrophilic nature of activated cisplatin (3). Therefore, the usage of antioxidants in combination with anti-cancer drugs is increasing for treatment in addition to their usage in prophylaxis of several diseases including cancer. A variety of polyphenolic compounds in plants are known as chemo-preventive antioxidant agents (4). Nowadays, epigallocatechin gallate (EGCG), a major component of polyphenols in green tea, becomes popular as an icon of antioxidants.

The polyphenolic catechin, EGCG, is believed to be the most protective agent in the green tea (5). EGCG has a potential to modify oxidative environments for tumor cell destruction and normal cell survival (6). In previous studies, EGCG were reported as a beneficial therapeutic agent for various types of cancers including lung, ovarian, breast, cervical, and prostate cancers (7–10) due to inhibiting cell proliferation, angiogenesis, and inducing apoptosis to prevent the growth of solid tumors (5). EGCG may also prevent the secondary malignancy and weight loss caused by cisplatin and/or tumors (11). The action of EGCG is believed to occur by its antioxidative properties. This catechin scavenges free radicals and prevents mutation and DNA damage by inducing expression of some enzymes such as glutathione peroxidase, glutamate cysteine ligase, and hemeoxygenase-1 (HO-1), which are all involved in the elimination of reactive oxygen species (ROS) (12). Furthermore, several studies showed that EGCG can also increase the sensitization of cancer cells to cisplatin even if they are resistant (13). EGCG is known to block many targets in signal transduction pathways (12). For example, EGCG tends a potent inhibitor of Akt/NF-κB and mTOR signaling pathways affecting apoptosis and cell survival mechanisms (14). Also, this catechin causes nuclear factor erythroid 2-related factor 2 (Nrf2)-mediated antioxidant induction and reduced inflammation (15). Nrf2, a redox-sensitive transcription factor, regulates transcriptional activation through the antioxidant response element (ARE) to prevent the oxidative stress. The activation of Nrf2 is considered an important molecular target of cytoprotective agents. Upregulation of Nrf2 by its activators resulted in an increased expression of antioxidant enzyme HO-1 (12). The action of Nrf2 on the prevention of inflammation enhanced by ROS occurs through the inactivation of nuclear factor-κB (NFκB) (16).

In one of our studies, we observed that EGCG supplementation improved the cisplatin-induced nephrotoxicity by reducing inflammation through NF-κB inactivation. Furthermore, in this study, we noted that Nrf2/HO-1 signaling pathway may be the primary target of EGCG for prevention of cisplatin-induced nephrotoxicity in a mouse model (15). Although there are several studies related with anti-proliferative and antioxidant activity of EGCG, there is limited number of published data on *in vitro* studies of molecular signaling mechanisms of EGCG on cervical cancer cell investigating only apoptotic pathways (17). Clearly, much more research is needed to determine which molecular mechanisms are responsible for the sensitization of the cancer cells by EGCG to chemotherapeutic drugs. Therefore, the goal of the current study is to investigate the possible molecular pathways to potentiate cervical cancer growth inhibition by combination therapy of cisplatin and EGCG *in vitro*. In this study, we evaluated the effects of EGCG and cisplatin on cell growth, apoptosis-related protein expression and oxidative stress in HeLa, and human cervical cancer cell line.

## Materials and Methods

### Cell culture and reagents

HeLa cells, the human cervical cancer cell line, were obtained from American Type Culture Collection (Manassas, VA, USA). They were maintained in RPMI-1640 medium containing 10% heat inactivated fetal bovine serum, 1% L-glutamine, 100 U/mL penicillin G, and 100 μg/mL streptomycin. Incubations of cells were done in a humidified, 5% CO_2_ atmosphere at 37°C. No growth factors were added to the cell culture medium. EGCG (Teavigo^®^, DSM, Istanbul, Turkey) was dissolved in 0.9% saline. Cisplatin (Sigma Chemical Company, St. Louis, MO, USA) was dissolved in phosphate buffered saline to make a 0.5 mM stock solution.

### Cell viability assay

Cell viability was determined by MTS Assays. HeLa cells were seeded 3000 cells in a 96-well plate and incubated overnight. Cells (2–5 × 104) were treated with EGCG (25 μM), cisplatin (250 nM), and their combination treatment for 24 h. After 24 h of total treatment, the cells were incubated at 37°C with 1 mg/mL MTS reagent (Sigma, St. Louis, MO, USA) for 2 h. The formazan crystals were dissolved in isopropanol. Spectrophotometric absorbance of the samples was determined by the ULTRA Multifunctional Microplate Reader (ELx800-BIO-TEK) at 490 nm. MTS assay repeated at least three times.

### Western blot analysis

The total protein extraction was performed from HeLa cells only, HeLa cells treated with EGCG (25 μM), cisplatin (250 nM), and EGCG co-treated with cisplatin for 24 h. Sample proteins were separated by sodium dodecyl sulfate polyacrylamide gels (SDS-PAGE) and subsequently transferred to a nitrocellulose membrane. Then, blots on the membrane were incubated with blocking solution (5% non-fat dry milk). Primary antibody was diluted (1:1000) in the same buffer containing 0.05% Tween-20. Afterwards, the blots were incubated overnight with primary antibody, anti-NF-κB p65, anti-COX-2, anti-HO-1, anti-p-mTOR, anti-p-p70S6K1, antip4E-BP1, anti-p-Akt (Abcam, Cambridge, UK), and anti-Nrf2 (Santa Cruz, CA, USA) at 40°C and on the following day, with secondary antibody (HRP-linked goat anti-mouse IgG, Abcam, Cambridge, UK) for 1 h at room temperature. Blots were then incubated with diaminobenzidine and H_2_O_2_ as substrates for visualization of specific binding. Protein loading was controlled using a monoclonal mouse antibody against β-actin (A5316; Sigma). Blotting was performed at least three times to confirm data reproducibility. Immunoreactive protein bands were quantified densitometrically using ImageJ analysis system (NIH, Bethesda, USA). Results were normalized to the β-actin expression in each group as percent of control. Blots were performed three times.

### Statistical analysis

Group means ± SEM were calculated from all measures, which were repeated at least three times. Data were analyzed by one-way ANOVA using the GLM Procedure (SPSS version 13.0, Chicago, IL, USA). The Tukey’s *post hoc* test option was employed to elucidate group mean differences. The criterion of statistical significance was *p* ≤ 0.05.

## Results

### EGCG enhances the inhibitory effect of cisplatin on the proliferation of HeLa cells

**Figure 1:**
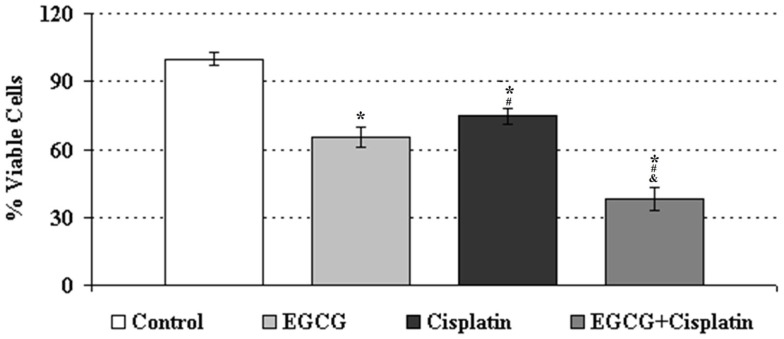
**Growth inhibition of human cervical cancer cell lines treated with EGCG (25 μM), cisplatin (250 nM), and the combination treatments**. *Indicates significant difference against to control, ^#^indicates significant difference against to EGCG, ^&^indicates significant difference against to cisplatin (*p* < 0.05). Error bars denote SEM.

MTS cell proliferation assay was used to evaluate the effects of EGCG, cisplatin, and their combination on the proliferation of HeLa cells. The relative cell viability for each treatment group was determined according to density of insoluble purple formazan dye products, which were formed due to reducing MTS by metabolically active cells (18). The individual treatment of cisplatin and EGCG decreased the growth of human cervical cancer cell lines to 74.7 and 65.5%, respectively. In the combination therapy, EGCG enhanced the inhibitory activity of cisplatin by decreasing the cell viability to 37.9% as compared to control group (Figure 1).

### EGCG enhances cisplatin-induced apoptosis in HeLa cells

**Figure 2:**
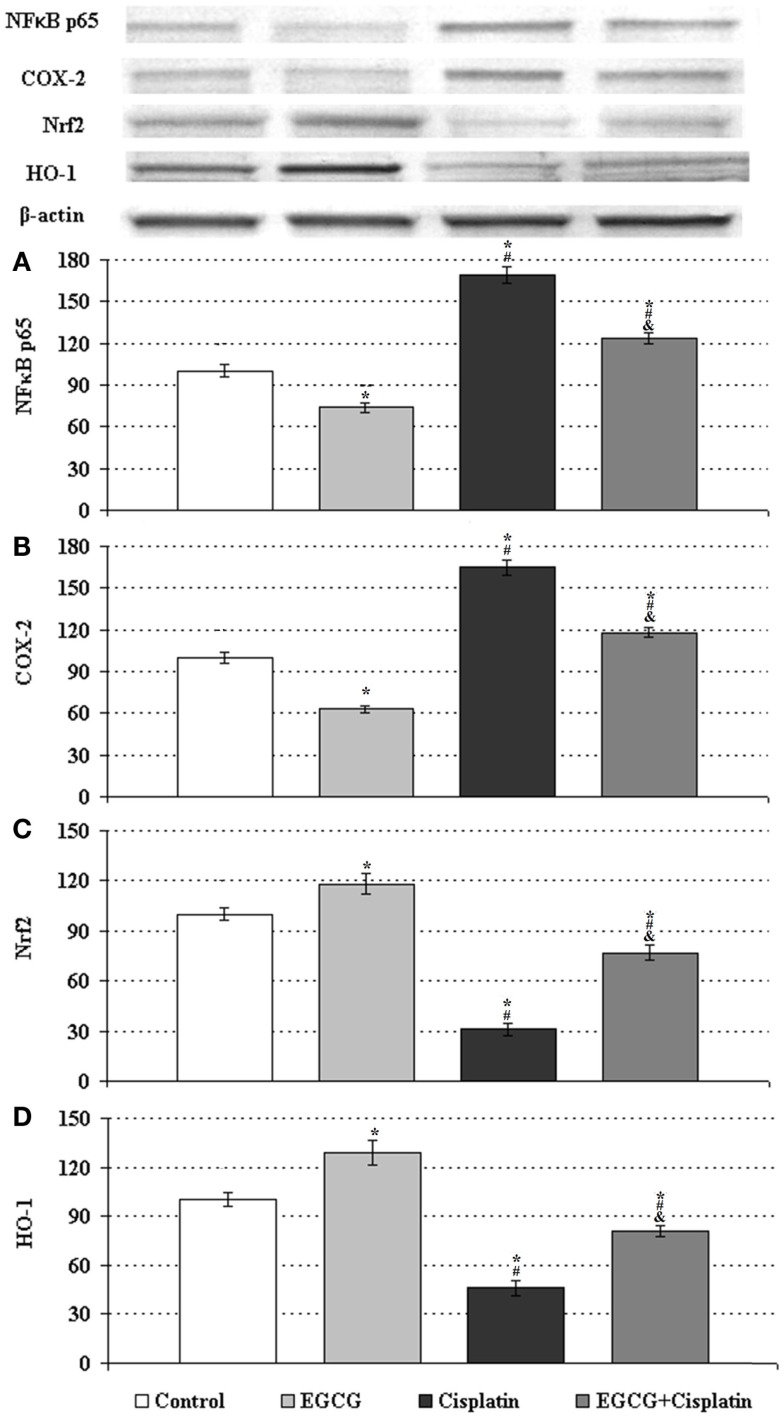
**Western blot analyses of NF-κB p65, COX-2, Nrf2, and HO-1 in HeLa cells**. The intensity of the bands for expression of **(A)** NF-kB, **(B)** COX-2, **(C)** Nrf2, and **(D)** HO-1 in HeLa cells were quantified by the densitometric analysis. Cells untreated or treated with 25 μM EGCG, 250 nM cisplatin (Cis), and the combination (EGCG + cisplatin). β-Actin antibodies were used as internal controls for equal loading of proteins. Data are percent of the control. *Indicates significant difference against to control, ^#^indicates significant difference against to EGCG, ^&^indicates significant difference against to cisplatin (*p* < 0.05). Error bars denote SEM.

Compared to control, cisplatin-only treated cells significantly increased the expression level of NF-κB p65B, whereas EGCG-only treated cells significantly decreased this protein level. When EGCG was applied to the cell culture with cisplatin, expression level of NF-κB p65B protein significantly reduced compared to cisplatin-only treated cells group (Figure 2A).

Western blot analysis showed that the expression of cyclooxygenase-2 (COX-2) was significantly higher in the cisplatin-treated HeLa cells than that of the control cells. The COX-2 expression was significantly decreased by EGCG-only treated cells and co-treated cells with cisplatin as compared with cisplatin-only treated cells (Figure 2B).

### EGCG reduces cisplatin-induced oxidative stress in HeLa Cells

To observe the role of EGCG in oxidative stress mechanisms, the expression of Nrf2 and HO-1 were evaluated by Western Blot analysis. The expression levels of Nrf2 and HO-1 significantly decreased in the cisplatin-only treated HeLa cells compared with those expressions in the control HeLa cells (Figures 2C,D). It was shown that EGCG increased Nrf2 and HO-1 accumulation in the nuclear fraction of EGCG-only treated cells compared to control cells. Combined treatment of cisplatin and EGCG was significantly increased these protein levels as compared to cisplatin-only treated cells.

### EGCG inhibits cisplatin-induced activation of mTOR pathway in HeLa cells

**Figure 3:**
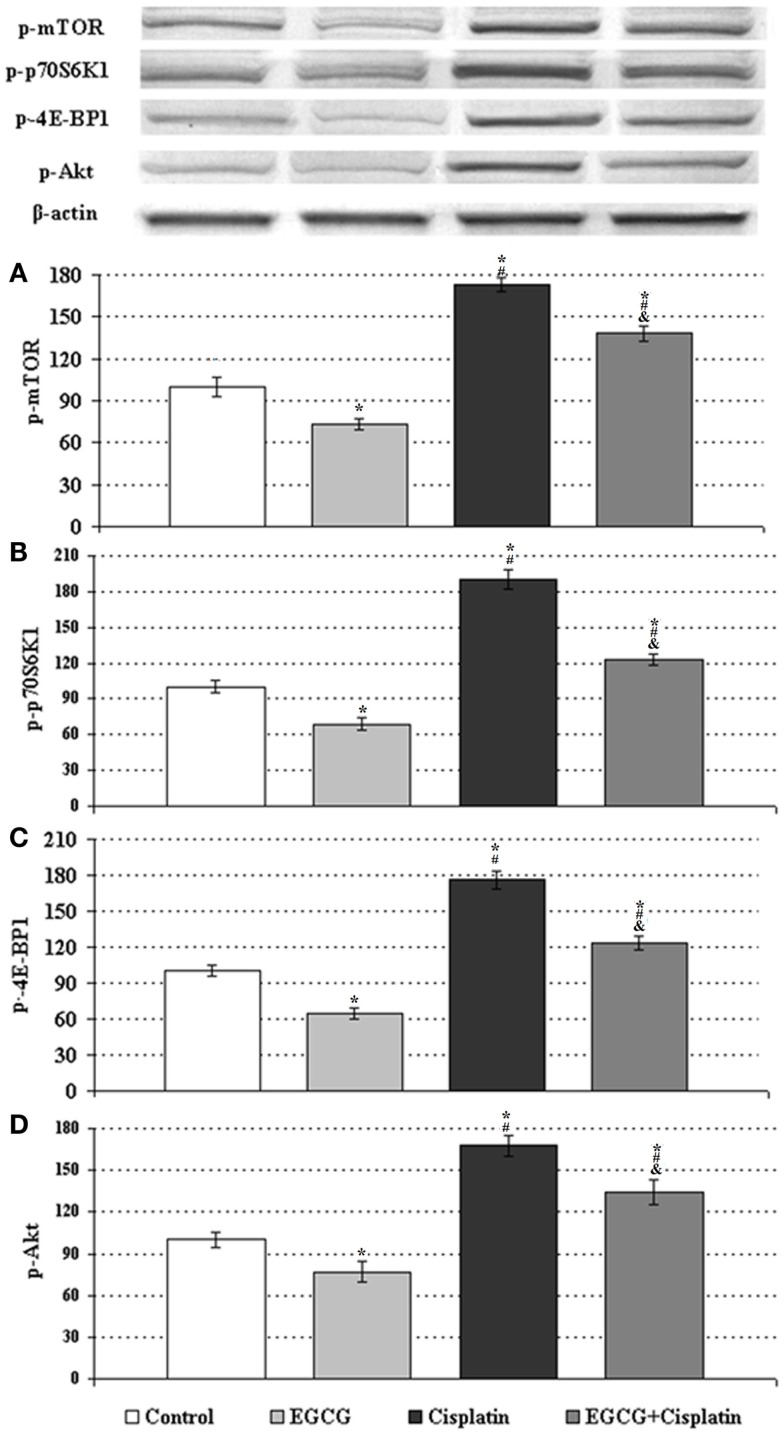
**Western blot analyses of p-mTOR, p-p70S6K1, p-4E-BP1, and p-Akt in HeLa cells**. The intensity of the bands for expression of **(A)** p-mTOR, **(B)** p-p70S6K1, **(C)** p-4E-BP1, and **(D)** p-Akt in HeLa cells were quantified by the densitometric analysis. Cells untreated or treated with 25 μM EGCG, 250 nM cisplatin (Cis), and the combination (EGCG + cisplatin). β-Actin antibodies were used as internal controls for equal loading of proteins. Data are percent of the control. *Indicates significant difference against to control, ^#^indicates significant difference against to EGCG, ^&^indicates significant difference against to cisplatin (*p* < 0.05). Error bars denote SEM.

In order to evaluate the involvement of mTOR molecular pathway in the anti-proliferative effect of cisplatin and ECGC in HeLa cells, we assessed the expression levels of p-mTOR, p-p70S6K1, p-4E-BP1, and p-Akt. Individual treatment of ECGC significantly reduced the level of phosphorylated mTOR, p70S6K1, 4E-BP1, and Akt in cervical cancer cells, while individual cisplatin treatment significantly increased their levels compared to control (Figures 3A–D). The combined therapy of EGCG and cisplatin attenuated the cisplatin-induced effects by decreasing the levels of p-mTOR, p-p70S6K1, p-4E-BP1, and p-Akt compared to cisplatin-only treated cells.

## Discussion

Cisplatin is a major chemotherapeutic agent for the treatment of cervical carcinomas as used in other cancer types. However, in addition to the efforts against to its toxicity, the amelioration of cisplatin sensitivity is an important point in treatment of cervical cancer. Besides, the usage of chemotherapy alone is generally ineffective against well-established tumors. Nowadays, additional substances such as antioxidants in combination with chemotherapeutic agents have been used to overcome the intrinsic resistance against therapy for tumors (19). Antioxidants have a dual effect in carcinomas. In addition to protection against cisplatin-induced toxicity, they also increase the sensitization of cancer cells to cisplatin (20, 21). Recently, the green tea polyphenol EGCG has gained attention as a natural agent for its chemo-preventive and anti-proliferative properties against various types of carcinomas. In the current study, we examined the action of EGCG on cisplatin-treated cervical cancer cells. We found that addition of EGCG to the cisplatin-treated cervical cancer cells attenuated the toxicity of cisplatin by decreasing cellular survival and inducing apoptosis.

The effect of EGCG on cisplatin’s chemotherapeutic activity has been previously reported for ovarian cancer (13), prostate cancer (22), and head and neck squamous carcinoma (23). In this study, compared to individual cisplatin treatment, we observed that combination treatment of EGCG and cisplatin significantly enhances the inhibition of cellular growth of cervical cancer cells *in vitro* as suggested in a recent study performed by Singh et al. (17). One of problem in the cancer chemotherapy is further increase the concentration of chemotherapeutic agent, thereby, increase in the cellular toxicity. Therefore, the sensitization of cisplatin-induced growth inhibition of HeLa cells by EGCG may be a remedy for chemotherapeutic drug toxicity-induced damages. The potentiation of cancer cell growth inhibition by EGCG was directed us to find the possible target pathways for how EGCG sensitize the HeLa cells to cisplatin.

Activation of mTOR signaling pathway is associated with cell survival in cervical cancer cells (21). mTOR is known to regulate initiation of translation through two pathways: S6K and 4E-BP1 (24). Herein, we have found increased p-mTOR, p-p70S6K1, and p-4E-BP1 expressions after cisplatin treatment, which downregulated by the addition of EGCG. These results suggest that the prevention of mTOR pathway upregulation occurs through p70S6K1 and 4E-BP1 proteins. Parallel changes were observed in the expression level of phosphorylated Akt suggesting presence of a crosstalk between mTOR pathway and Akt pathway in the sensitization mechanism of EGCG. The activation of Akt has a role in the development of chemotherapeutic drug resistance and reduction of the apoptotic-potential of chemotherapeutic agents in several cancer types (17). Therefore, the inhibitor agents of the Akt pathway can improve cisplatin-induced chemotherapy (17). EGCG is also reported as a potent inhibitor of Akt signaling pathway (14). Thus, inducing apoptosis by regulating Akt pathway may be a mechanism for inhibition the growth of cervical cancer cells.

Cisplatin-induced oxidative stress was previously reported in different tissues (20, 25). It has been shown that cisplatin triggers its toxicity by enhancing ROS production and TNF-α level, reducing the levels of antioxidant enzymes, and inducing apoptosis (25). One of the molecular target of cisplatin-induced oxidative stress is Nrf2/HO-1 pathway (12, 20). Nrf2 protects the cell against oxidative stress by inducing of several phase 2 detoxifying and antioxidant enzymes, such as HO-1. In this study, we observed that expression levels of Nrf2 and HO-1 reduced significantly in the cisplatin-only treated cells compared to control HeLa cells demonstrating the cisplatin cytotoxic activity on cancer cells through increasing oxidative stress. However, the combined therapy of cisplatin and EGCG increased Nrf2 and HO-1 expressions in the cervical cancer cells compared to cisplatin-only treated cells. This result suggested that EGCG activates Nrf2 pathway due to the effect of electrophiles induced by cisplatin. Previous studies demonstrated that overexpression of Nrf2/HO-1 confers resistance to apoptosis induction by EGCG (26). Under physiological conditions, cytosolic Nrf2 is inactive by its negative regulator Kelch-like ECH-associating protein 1 (Keap1). The EGCG’s action on Nrf2 pathway might be due to its interaction with Keap1 thiols. When cells are exposed to redox modulators, in this case EGCG treatment, Nrf2 is released from Keap1 protein and hence translocates and accumulates in the nucleus acting as an electrophiles and pro-oxidant stressors sensor (27). The activation of Nrf2 may transcriptionally regulate an antioxidative gene, HO-1 (12). These results suggest that EGCG may stimulate the synthesis of antioxidant systems using Nrf2 pathway. The EGCG’s cytoprotective mechanism by activating Nrf2-mediated antioxidant response is best demonstrated in animal studies (12, 15, 27). However, in cancer cells, the overexpression of Nrf2/HO-1 by EGCG promotes the survival of cancer cells under a deleterious environment such as cisplatin-induced oxidative stress. This condition decreases Keap1 expression and upregulates the Nrf2 expression resulting in removal of ROS and protection of cancer cells (28).

The oxidation and nitration of another oxidative stress sensitive molecular target, transcription factor NF-κB, which is induced by Nrf2-driven transcriptional activation of ARE, has an essential role in the oxidant/redox signaling (16). Therefore, various physiological and pathological stimuli like ROS activate this protein. NF-κB also plays an important role in apoptosis mechanisms by exerting its regulatory effects on survival genes in addition to its role in inflammation (16). Similar to previous observations, in the current study, cisplatin treatment significantly increased NF-κB p65 protein level. It was shown that disruption of Nrf2 enhances the upregulation of NF-κB (26) in agreement with our study. Also, activated NF-κB has also been identified as a key mechanism of cisplatin resistance (29). Previous studies done in different cancer cell lines, like epidermoid carcinoma cells and head and neck squamous cell carcinoma, showed that upregulation of NF-κB activation can result cisplatin resistance either by influencing expression of Bcl-2 family members or MAPK signaling pathways and regulating chromatin remodeling (30, 31). EGCG treatment downregulated the increase in the expression level of NF-κB p65 induced by cisplatin as compared to individual cisplatin treatment pointing the potent antioxidative capacity of EGCG. The decrease of this protein expression in cancer cells by plant extracts is also supported by our previous studies (21) and other researchers (26).

COX-2, an NF-κB target gene, is expressed at high levels in pathological circumstances and oncogenesis (32). In carcinogenesis, COX-2 is related with abnormal arachidonic acid metabolism due to its role in biosynthesis of prostaglandins through the conversion of arachidonic acid. Parallel to NF-κB expression, the protein expression of COX-2 was higher in cisplatin-only treated HeLa cells whereas EGCG-only treated cells and co-treated cells decreased COX-2 level compared to control and cisplatin-only treated HeLa cells. Given that the COX-2 is associated with (32), it is proposed that the downregulation of COX-2 by EGCG may have beneficial effects on reversing the cisplatin-induced apoptosis inhibition, thus, offering protection against cancer development (33). These results possibly suggested that EGCG’s chemo-preventive activity may occur through suppression of COX-2 target gene expression by inhibiting NF-κB to stop proliferation of cancer cells.

Herein, we have demonstrated that EGCG works synergistic with cisplatin in inhibiting growth of cervical cancer cells. Our findings suggest that cisplatin treatment is potentiated with EGCG in HeLa cells by regulating NFκB p65, Akt, and mTOR pathways, which are critical for cell survival and apoptosis. Collectively, our observations reveal that cisplatin and EGCG combination could be used to improve the treatment in cervical cancer through decreasing cell survival and enhancing apoptosis. This combination is a less toxic option in the treatment of cervical cancer, especially if there is chemoresistance to cisplatin. Therefore, this study may give a hint at chemotherapeutic uses of EGCG. Future *in vivo* and *in vitro* inhibition studies are warranted to investigate the combination of cisplatin and EGCG in cervical cancer when considered the high incidence rate of cervical cancer and associated side-effects of current treatment modality.

## Authors Contributions

The project was designed and implemented by Ulkan Kilic and Kazim Sahin. Data were analyzed by Mehmet Tuzcu, Nazli Basak, Cemal Orhan, Birsen Elibol-Can, Ertugrul Kilic, Fikrettin Sahin, and Omer Kucuk. Ulkan Kilic prepared the manuscript. Ulkan Kilic and Kazim Sahin supervised overall project. All authors read and approved the final version of manuscript.

## Conflict of Interest Statement

The authors declare that the research was conducted in the absence of any commercial or financial relationships that could be construed as a potential conflict of interest. The reviewer Mustafa Ozen declares that, despite having collaborated with the author Fikrettin Sahin, the review process was handled objectively and no conflict of interest exists.
